# Phylogeny and Body Size Predict Distress Call Divergence in Bats: A Comparative Analysis

**DOI:** 10.3390/ani15223268

**Published:** 2025-11-12

**Authors:** Yujuan Wang, Xiaobin Huang, Kangkang Zhang, Lixin Gong, Hao Gu, Wentao Dai, Jiang Feng, Tinglei Jiang

**Affiliations:** 1 Jilin Provincial Key Laboratory of Animal Resource Conservation and Utilization, Northeast Normal University, 2555 Jingyue Street, Changchun 130117, China; wangyj426@nenu.edu.cn (Y.W.); zhangkk307@nenu.edu.cn (K.Z.); gonglx216@nenu.edu.cn (L.G.); guh550@nenu.edu.cn (H.G.); daiwt930@nenu.edu.cn (W.D.); fengj@nenu.edu.cn (J.F.); 2 Yunnan Provincial Key Laboratory for Zoonosis Control and Prevention, Institute of Pathogens and Vectors, Dali University, Dali 671000, China; 3 College of Life Science, Jilin Agricultural University, 2888 Xincheng Street, Changchun 130118, China

**Keywords:** bat, distress calls, evolutionary divergence, comparative analysis

## Abstract

The evolutionary divergence of animal vocalizations is a complex process shaped by various factors, including morphology, ecology, social pressure, and phylogenetic relationships. This applies even to distress calls, which may appear to be simple expressions of emotion. However, the relative importance of these factors in shaping interspecific divergence of distress call structure remains largely unexplored. In addition, previous studies concerning the factors driving distress call divergence have been mainly conducted in birds, anurans, and lizards, whereas less is known for mammals. Here, we recorded distress calls across 32 bat species belonging to 7 families, and compiled data on body size, foraging habitat types, climatic variables, colony size, and phylogenetic components, aiming to identify the key determinants predicting the cross-species divergence in the incidence and acoustic parameter of distress calls within a comparative framework. Our results supported that bats divergence in distress vocalizations is not an adaptive evolution in response to ecological and social pressure, but constrained by phylogenetic relationship and body size. Phylogenetic components explained the most interspecific variation in the incidence and temporal parameters of distress calls, whereas body size accounts for spectral parameters. This study has revealed that distress vocalizations not only convey the caller’s emotional state, but also suffer severe phylogenetic and morphological constraint.

## Simple Summary

Bats often emit loud distress calls when captured or threatened, which can function to startle predators or alert conspecifics. However, little is known about how these calls vary among species and what factors drive such divergence. In this study, we recorded distress calls from 32 bat species in China and examined how evolutionary history, body size, ecology, and social traits shape call structures. We found that both phylogeny and body size were the main factors explaining differences in distress calls across species, while ecological and social factors played minor roles. These findings indicate that distress calls are not merely emotional outbursts but are shaped by deep evolutionary and morphological constraints, providing new insights into the evolution of acoustic communication in mammals.

## 1. Introduction

Animal vocalizations are well-known as an adaptive trait suffering various selective forces [1,2,3], and this is true even for distress calling that may seem like a simple expression of emotional state [3,4,5]. Distress calls (also called fear screams) are a specific type of alarm signals, and are produced by a broad range of animal taxa when confronted with imminent risk of death from predators [4]. These calls usually exhibit harmonic, broadband and noisy structures that propagate efficiently and are easily localized [6,7]. Otherwise, distress calls may serve as signals of body condition [8], and have multiple adaptive functions explained by several non-mutually exclusive hypotheses [7], including startling predators [9], attracting another predator [10], warning kin of the presence of a predator [3,11,12], and requesting aid from conspecifics and heterospecifics [13].

However, not all species, nor all individuals of a species, give distress calls. Instead, there is a significant interspecific variation in the tendency to scream. Previous studies have shown that when birds are cornered, attacked, or captured by predators, the incidence of distress calling varies greatly among species, ranging from 0 to 100% [14,15]. Similarly, remarkable interspecific differences in the percentage of individuals emitting distress calls have also been documented in frogs [16]. The propensity to emit distress calls is usually treated as an adaptive trait related to body size, habitat types, and colony size [3]. For example, Neudorf and Sealy [14] showed that larger bird species had a higher incidence of distress calling than smaller birds. Hogstedt [17] found that the propensity to produce distress calls differed among bird species, depending not only on body size but also on their habitat types, such that species living in closed habitats call more than species occupying open habitats. Moreover, flocking bird species do exhibit a significantly higher proportion of individuals emitting distress calls than solitary species do [18].

Vocal structure variation across species is considered the result of interactions of different selective pressures and constraints working on their production, transmission, and detection [19]. For instance, some researchers stress the importance of ecological selection, which suggests that species-level acoustic divergence is directly influenced by ecological factors relevant to sound degradation and attenuation, such as habitat features [20] and climatic variables [21]. Alternatively, mounting evidence has shown that morphological constraint significantly explains the interspecific variation in acoustic signals. A negative allometry between body size and frequency of vocalizations often is found in anurans [22], birds [23], and mammals [24]. Unsurprisingly, animal vocalizations can exhibit a certain degree of phylogenetic conservativeness, thus more similar acoustic signals are found between closely related species, such as frog advertisement calls [25], gibbon duets [26], and bird songs [27]. Furthermore, sociality has been proposed to explain the acoustic divergence between species recently. Species living in larger groups give comparatively broadband calls to increase individuality signature information due to stronger selective pressure on individual recognition [19,28]. Earlier studies on distress call structure in birds and lizards have revealed that distress vocalizations suffer severe morphological constraint, and larger species produce calls exhibiting lower frequency features and longer duration [29,30]. However, these studies focused mainly on body size; therefore, the relative strength of different factors (e.g., morphology, ecology, social pressure, and phylogenetic relationships) underlying cross-species acoustic divergence of distress calls remains unknown.

To date, much work on the factors explaining the interspecific variation in distress calling has involved birds, anurans, and lizards [30,31], while comparatively little information is available for mammals. Bats represent an interesting mammal group that relies heavily on acoustic signals for navigation and social communication in darkness. Moreover, bats are recorded from all areas of the world except the polar regions and a few oceanic islands [32], and exhibit a wide range of body sizes (from 2 g to 1.2 kg) [33], foraging habitats (including open, edge, and narrow space habitats) [34], and social group sizes (from a few individuals to millions) [35]. Hence, bats provide an excellent opportunity to explore how different factors shape interspecific divergence in vocal behavior in mammals.

In the present study, we recorded distress calls from 32 bat species belonging to 7 families, and compiled a data set including body size, habitat types, annual mean temperature, annual precipitation, phylogenetic components, and estimated colony size of species. We aim to elucidate the key factors underlying interspecific divergence in the incidence and acoustic characteristics of distress calls within a comparative framework. If morphological factors contribute to distress calls divergence, we predicted that bat body size would be positively associated with the propensity to scream, but negatively correlated with the frequency attributes of vocalizations [29,36]. If ecological factors drive vocal variation among species, the percentage of individuals emitting distress calls should vary with habitat type, and acoustic features should be significantly influenced by habitat types and climatic variables [17,20]. If sociality is responsible for the interspecific divergence of vocal behaviors, species living in larger colonies would have higher probability to give distress calls with wider bandwidth [18,19]. If distress calling suffers phylogenetic constraint, a robust correlation between phylogenetic components and the incidence and acoustic parameters of distress calls would be expected [2].

## 2. Materials and Methods

### 2.1. Study Species and Sites

**Figure 1:**
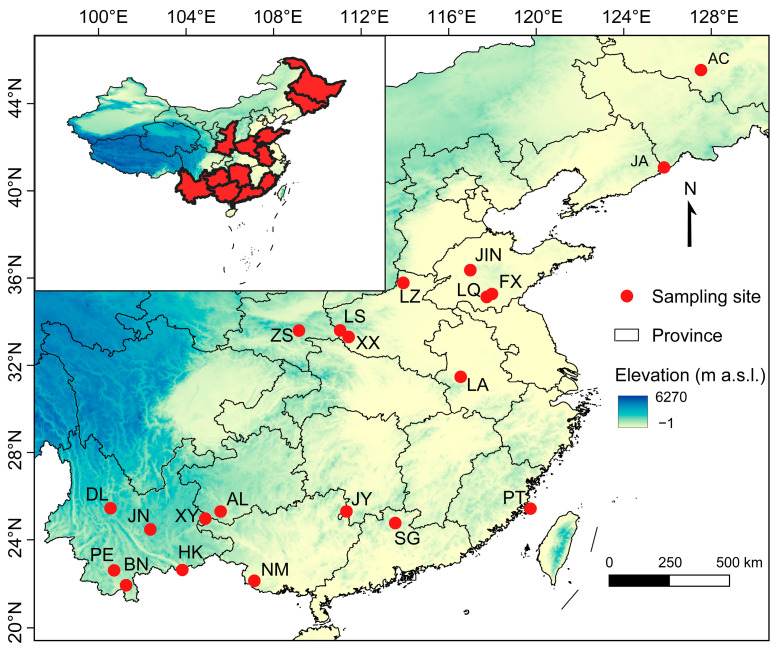
Localities for distress call recording for 32 bat species in China. Information on site abbreviations and bat species are provided in the Supplementary Material Table S1.

From 2011 to 2016, we collected 623 adult bats from 32 species belonging to 7 families, including Pteropodidae, Hipposideridae, Rhinolophidae, Vespertilionidae, Miniopteridae, Molossidae, and Emballonuridae (Table S1). All bats were captured in their roosts using mist nets or hand nets at 21 different localities in China (Figure 1), and were then taken into a temporary laboratory near the roosts. Captured bats were identified to species, sexed, and aged, and their reproductive status was also assessed based on external characteristics. Subadults and pregnant or lactating females were released immediately. Forearm length was measured using digital calipers (TESA-CAL IP67, TESA Tech., Renens, Switzerland). Then, we collected a small piece of wing membrane tissue (bat wing punctures) for subsequent genetic analysis at Northeast Normal University (NENU). All procedures were approved by the Animal Ethics Committee of Northeast Normal University (approval number: NENU-2015-05) and conformed to the ethical guidelines for animal use.

### 2.2. Recording and Analysis of Calls

Distress calls were recorded using an UltrasoundGate 116 (Avisoft Bioacoustics, Berlin, Germany) connected to a laptop computer, with an appropriate sampling frequency (250, 375, or 500 kHz) and 16-bit resolution. The distance between the bat and the microphone was approximately 1 m. To encourage calling, we held bats in the hand and gently massaged their lower back for 2 min. Each bat was handled by the same person. All bats were released at the site where they had been captured after data collection. The acoustic analyses were carried out with Avisoft SASLab Pro software version 5.2.12 (Avisoft Bioacoustics, Berlin, Germany). Spectrograms were generated using a 512-point fast Fourier transform (FFT) and a Hamming window with 100% frame size and 93.75% overlap (frequency resolution = 488 Hz, time resolution = 0.128 ms).

To capture interspecific variation in vocal behaviors, we first measured the incidence of distress calling for each species (the percentage of callers in a given species). During the two-minute handling, an individual who emitted at least one distress call was assigned as “caller”, and who remained silent was assigned as “non-caller” (Table S2). Sex differences in the percentage of individuals emitting distress calls were not significant in bats based on our analysis (Pearson’s chi-square test: all *p* > 0.05), and no significant differences were detected in acoustic parameters (all *p* > 0.05). Thus, we combined data from males and females, and included 30 species with sample sizes of 7 or more individuals in further statistical analysis for the incidence of distress calling (Table S1). Then, we selected distress calls recorded during handling with high signal-to-noise ratio, and measured 7 acoustic parameters (Figure S1; Table S2), including syllable rate (syllable/sec) and 6 parameters of distress noises (defined as the syllable that shows no clear fundamental frequency and no clear harmonic structure), which represent the most common syllable type in bat distress calls [6,37]. The following acoustic parameters were measured from distress noises: syllable duration (the time between the onset and end of a noise syllable; ms), fundamental frequency (the frequency of the first harmonic peak; kHz), peak frequency (the frequency at maximum amplitude; kHz), minimum frequency (−20 dB below peak frequency; kHz), maximum frequency (−20 dB below peak frequency; kHz), and bandwidth (maximum frequency minus minimum frequency; kHz).

### 2.3. Data Collection

Forearm length does not vary with food intake or season in bats compared with body mass and, therefore, was selected as an indicator of body size to assess the effect of morphological constraints on vocal behavior [38]. We used foraging habitat types and climatic variables to describe ecological factors relevant to sound degradation and attenuation. Foraging habitats of each bat species were classified into three categories, namely open, edge, and closed habitats based on field survey, acoustic monitoring, and information from previous ecological studies and databases (the habitat types are defined according to the bats’ echolocation behavior in relation to the distance between bat and background or food item and background; Table S1) [19,34,39,40]. We downloaded the global distribution range map for each bat species from the IUCN Red List database [40], and overlaid it with the WorldClim bioclimatic rasters (version 2.1; 1970–2000; 2.5 arc-min) to extract the annual mean temperature (BIO1) and annual precipitation (BIO12) experienced by each bat species using the “raster” R package (version 3.5-15). In addition, we amplified cytochrome b (Cytb) sequences from wing tissues of each species via polymerase chain reaction (PCR). Among these samples, some sequences have been reported in Luo et al. [19]. All sequences were aligned using Clustal X2 (version 2.1) and modified with manual adjustments (GenBank accession number of species were listed in Table S1). Then, we constructed a genetic distance matrix based on Cytb sequences, and performed principal coordinate analysis (PCoA) to transform genetic distances to eigenvectors, using the “ape” package (version 5.0) [41]. We extracted the first two axes of the PCoA (explained ~50% of genetic distance among species) as the proxy for phylogenetic components. Although phylogenetic generalized least squares (PGLS) is often used to control for phylogenetic effects, it cannot treat phylogeny as a quantitative predictor in multivariate analyses. Therefore, we used a PCoA-based approach that enabled direct integration of phylogenetic information into the statistical framework to assess the relative importance of multiple factors. Group size is usually treated as a classic indicator of social pressure [28]. Thus, we estimated each species’ colony size following the method adopted by Luo et al. [19], or using the mark-recapture method in *Cynopterus sphinx* [42].

### 2.4. Statistical Analyses

Chi-squared test was performed to examine the interspecific variation in the percentage of individuals emitting distress calls using the function “prop.test”. Then, we employed a linear mixed model analysis (LMM) to explore the determinants driving the variation in incidence of distress calls using the “lme4” package (version 1.1-37) [43]. The incidence of distress calls for each species, calculated as the number of callers divided by the total number of individuals captured (Table S2), was assigned as the response variable and square-root transformed to approximate a normal distribution. Foraging habitat, colony size, forearm length, and phylogenetic components were entered as predictor variables, while sampling location was entered as a random effect.

Using all acoustic parameters per species, we conducted a permutational multivariate analysis of variance (PMANOVA) to inspect species-level vocal structure variation using the package “vegan” (version 2.7-2) [44]. Subsequently, we calculated the mean value of all call parameters per species, and reduced the dimensionality of calls by means of a principal component analysis on the mean acoustic parameters of all species using the “Psych” package (version 2.5.6) [45]. Then, we performed another linear mixed model analysis to assess the effects of different factors on acoustic parameter variations in distress calls. The extracted principal components were assigned as response variables and were normally distributed (Kolmogorov–Smirnov test, all *p* > 0.05). Habitat types, colony size, forearm length, phylogenetic components, annual mean temperature, and annual precipitation were included as predictor variables, with sampling site as a random effect.

Model selection for both LMMs was conducted based on the Akaike Information Criterion corrected for small sample size (AICc) using the “dredge” function in the MuMIn package (version 1.46.0) [46]. Competing models were ranked according to their differences in AICc scores (ΔAICc), and models within ΔAICc ≤ 2 of the best model were considered strongly supported. When multiple models met this criterion, model averaging was performed using the “model.avg” function in MuMIn [46] to obtain averaged parameter estimates and 95% confidence intervals (95% CIs) for all predictors included in the supported models. A predictor was considered to have a significant effect on variation in distress calls when its 95% CI did not overlap zero. If only one model satisfied ΔAICc ≤ 2, model averaging was unnecessary, and variables retained in that model were regarded as significant.

To assess the independent explanatory power of each predictor variable for interspecific variation in both the incidence and acoustic characteristics of distress calls, we conducted hierarchical partitioning analysis using the “hier.part” package (version 1.0-6) [47]. Statistical significance for each variable was determined through randomization testing [48]. Variables supported by both model averaging and hierarchical partitioning analyses were identified as key factors explaining interspecific variation in bat distress calls [8]. All statistical analyses were run in R 4.3.3.

## 3. Results

### 3.1. Incidence of Distress Calling in Bats

**Figure 2:**
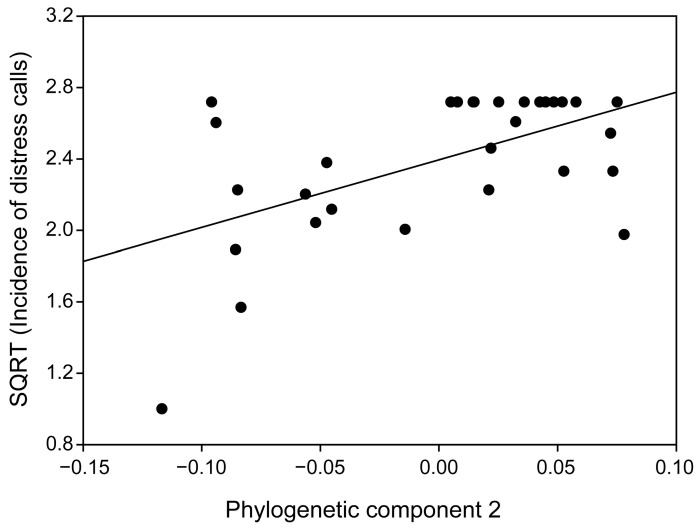
Relationship between the proportion of individuals emitting distress calls and the second phylogenetic component. Each black dot represents a different species.

The proportion of individuals emitting distress calls varied from 0 to 100% among bat species (Table S2), and exhibited significant interspecific variation (Chi-square test: *χ*^2^ = 180.33, *df* = 29, *p* < 0.001) while being handled. Apart from Pteropodidae and Emballonuridae, most species had high incidence of distress calling (>66.00%; Table S2). Model selection revealed that only one model met the ΔAICc ≤ 2 criterion (Table 1). Habitat types, colony size, and forearm length were not retained in this best-supported model, whereas the second phylogenetic component was identified as the sole significant predictor (Table 1). The hierarchical partitioning approach further supported this result, showing that the second phylogenetic component had a significantly higher independent explanatory power for interspecific variation in the incidence of distress calls (phylogenetic component 2: *p* < 0.05, 52.20%; other predictor variables: all *p* > 0.05, 1.20–22.04%). Thus, a robust relationship between phylogenetic component and the tendency to emit distress calls was found (Figure 2).

### 3.2. Distress Call Structure

**Figure 3:**
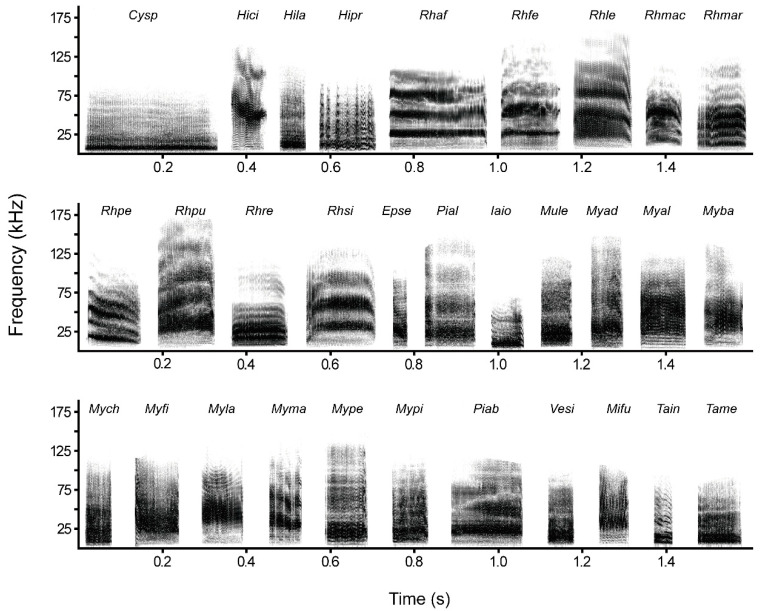
Spectrograms of bat distress calls. *Cysp*: *Cynopterus sphinx*. *Hici*: *Hipposideros cineraceus*. *Hila*: *Hipposideros larvatus*. *Hipr*: *Hipposideros pratti*. *Rhaf*: *Rhinolophus affinis*. *Rhfe*: *Rhinolophus ferrumequinum*. *Rhle*: *Rhinolophus lepidus*. *Rhmac*: *Rhinolophus macrotis*. *Rhmar*: *Rhinolophus marshalli*. *Rhpe*: *Rhinolophus pearsonii*. *Rhpu*: *Rhinolophus pusillus*. *Rhre*: *Rhinolophus rex*. *Rhsi*: *Rhinolophus sinicus*. *Epse*: *Eptesicus serotinus*. *Pial*: *Pipistrellus alaschanicus*. *Iaio*: *Ia io*. *Mule*: *Murina leucogaster*. *Myad*: *Myotis adversus*. *Myal*: *Myotis altarium*. *Myba*: *Myotis badius*. *Mych*: *Myotis chinensis*. *Myfi*: *Myotis fimbriatus*. *Myla*: *Myotis laniger*. *Myma*: *Myotis macrodactylus*. *Mype*: *Myotis pequinius*. *Mypi*: *Myotis pilosus*. *Piab*: *Pipistrellus abramus*. *Vesi*: *Vespertilio sinensis*. *Mifu*: *Miniopterus fuliginosus*. *Tain*: *Tadarida insignis*. *Tame*: *Taphozous melanopogon*.

Bat distress calls exhibited a harmonic and noisy structure with dominant energy at low frequencies (Figure 3). Distress calls given by bats also showed notable interspecific variation in spectro-temporal parameters (PMANOVA: *F* = 52.05, *df* = 30, *p* = 0.001). Principal component analysis produced two principal components accounting for 82.58% of the variation in the original data set. The first principal component (PC1) positively related to all spectral parameters, namely fundamental frequency, peak frequency, minimum frequency, maximum frequency, and bandwidth. The second principal component (PC2) captured variation in temporal parameters, and was positively associated with syllable rate and negatively with syllable duration (Table 2; Figure S2).

**Figure 4:**
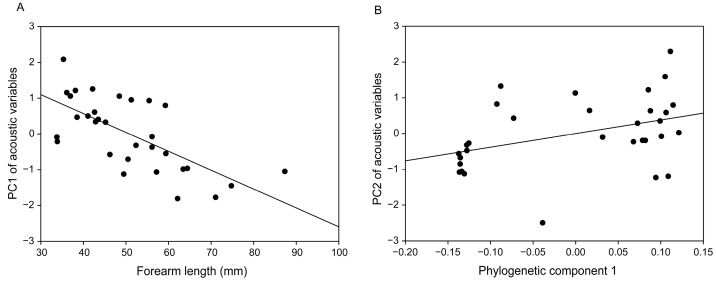
Relationships between (**A**) forearm length and the first principal component (PC1) of acoustic variables, and (**B**) the first phylogenetic component and the second principal component (PC2) of acoustic variables in bat distress calls. Each black dot represents a different species.

Model averaging revealed that forearm length, colony size, and the first phylogenetic component were significantly associated with PC1 (Table 3), which primarily represented spectral parameters. However, hierarchical partitioning identified only forearm length as a significant predictor. Forearm length independently explained a much larger proportion of the variation in PC1 than the other predictors (forearm length: *p* < 0.05, 69.95%; other: all *p* > 0.05, 0.65–11.54%; Table 3). These results indicate that spectral variation in bat distress calls is mainly driven by morphological constraints, with larger bat species tending to produce distress calls characterized by lower spectral frequencies (Figure 4).

For PC2, which reflected temporal parameters, model averaging indicated significant associations with both the first and second phylogenetic components (Table 3). However, hierarchical partitioning only supported the effect of the first phylogenetic component, which independently explained a higher proportion of the variation in PC2 than the other predictors (phylogenetic component 1: *p* < 0.05, 58.18%; other: all *p* > 0.05, 1.48–21.89%; Table 3). Therefore, these results support that temporal variation in bat distress calls is predominantly shaped by phylogenetic relatedness (Table 3; Figure 4).

## 4. Discussion

Our comparative analysis focused on the factors predicting distress call divergence in bats and obtained two important results. First, the propensity to emit distress calls was closely correlated with phylogenetic components. Second, body size and phylogenetic relationship explained the most acoustic variation in spectral and temporal parameters. Together, our findings demonstrated that phylogeny and morphology, rather than ecological or social factors, play dominant roles in shaping the evolutionary divergence of distress calls among bat species.

### 4.1. Incidence of Distress Calling

To maximize the expected fitness, prey species employ a variety of escape tactics when captured by a predator [49]. One such tactic is to produce distress calls. However, besides increasing the probability of escape of prey, distress calls also can provoke more vigorous attacks from predators and increasing the caller’s probability of dying [3,11]. This occurs because distress calls can signal that the prey is not yet fully restrained, prompting predators to kill it immediately to prevent escape [9]. Consequently, animals may adjust calling propensity based on body size, habitat types, and colony size to maximize survival chances. For instance, larger prey species are less likely to be killed immediately by predators and often require more time and effort to be fully subdued [29]. Therefore, the risk associated with distress calling is relatively lower for them, and their louder calls may also more effectively startle predators [14]. As a result, larger prey species are more inclined to produce distress calls to maximize their survival benefits, whereas smaller species are more likely to exhibit feigning death—a defensive strategy that also occurs in many taxa but is more common in smaller species that are less capable of physical resistance or startling predators [3,29,50]. Furthermore, species inhabiting dense habitats may call more frequently to attract other individuals (e.g., conspecifics and secondary predators) that can distract the original predator than species inhabiting open habitats where predation events can be detected visually [3,17]. Several hypotheses suggest that distress calls may warn conspecifics of the presence of a predator, or elicit aid from nearby individuals, thus gregarious species usually had a higher incidence of distress calling compared with solitary species [18].

Surprisingly, we did not find any significant effects of body size, habitat type, and colony size on the propensity for distress calling in bats. This result can be explained by three possible reasons. First, bats’ nocturnal habits, group living, flight, and echolocation ability deter or minimize the possibility of being attacked by potential predators [51]; therefore, additional behavioral adaptations to increase individual survival appear to be unnecessary. Second, incidence of distress calling may be limited by the night vision of bat species which may mute the role of other factors. Some studies have shown that bats prefer to use visible signal rather than sonar cues when both acoustical and visual cues are available [52]. Chiroptera, Pteropodidae, and Emballonuridae have more developed vision for navigation than other bat families in the darkness [53]. Indeed, *Rousettus leschenaultia* and *C*. *sphinx* belonging to Pteropodidae, and *Taphozous melanopogon* belonging to Emballonuridae exhibited lower propensities to emit distress calls compared with species from other bat families. However, more work is required to test this explanation because of our fairly scarce knowledge of the night vision of bat species. Third, trophic guild may also play an important role in shaping the likelihood of distress calling. In our study, the frugivorous species *R. leschenaultii* and *C. sphinx* showed relatively low probabilities of distress calling, whereas most insectivorous bats—such as species in *Rhinolophus* and *Myotis*—displayed nearly 100% incidence of distress calling (Table S2). Such trophic differences may indirectly weaken the effects of other ecological or social factors on calling behavior. Nevertheless, because of the limited sample size for non-insectivorous trophic categories, we cannot conclusively determine whether trophic ecology is the primary factor driving this difference. Future comparative studies incorporating a broader diversity of dietary guilds are needed to further evaluate this possibility.

Intriguingly, we found that phylogenetic components significantly explained the variation in incidence of distress calling among bat species. Even if numerous studies have shown that morphological, behavioral, life-history, and ecological characteristics in different taxa exhibit conserved in phylogeny [54,55], ours is the first study that demonstrates a pronounced relationship between the propensity for calling and phylogenetic relatedness. Widespread phylogenetic characters across taxa usually result from their genetic inheritance from common ancestors [56]. In addition, we also cannot rule out an indirect effect, thus phylogenetic relatedness may affect body size, social factors, ecological niches, and night vision, and then the incidence of distress calling indirectly [19,55]. In our study, bat species belonging to the same family showed more of a similar tendency to scream, and species in Rhinolophidae exhibited the highest proportion of individuals emitting calls.

### 4.2. Structure of Distress Calls

Distress calls are given at the last stage of a predator/prey interaction when prey are in considerable danger and fear [13]. These calls are usually described as harsh (i.e., spreading call energy over a wider range of frequencies) and low-frequency sounds with a broadband and noisy structure in a broad range of taxa [57]. In the present study, a similar acoustic design was found in distress calls of bats. Motivation–structural rules uncover a general relationship between the physical structures of sounds and the motivation state, which suggest that birds and mammals tend to emit harsh, low-frequency calls in hostile-aggressive situations [5,58]. Furthermore, Naumann and Kanwal [59] found that broadband noise obviously excited neurons within the receivers’ basolateral amygdala, which is centrally involved in encoding and expression of fear. Hence, distress call structure can effectively convey an emotional state of fear and aggression.

Animal vocalizations arise from the vibrations of sound-producing apparatus whose size are usually strongly and significantly correlated with body size [19,60]. In general, large animals tend to have larger larynges, longer vocal tracts, or thicker syringeal membranes, and thus produce lower pitched calls [61]. Morphological constraint has been considered as a vital factor to shape species-level acoustic divergence in most vertebrates. For instance, Gingras et al. [62] confirmed a tight and widespread negative correlation between snout-vent length and call frequency in 136 species belonging to four clades of anurans. Similarly, Bowling et al. [63] showed an inverse relationship between body length and vocalization frequencies in 91 species of primates and carnivores. Our results also revealed a negative size–frequency allometry across bat species, and spectral parameters of distress calls (i.e., fundamental frequency, peak frequency, minimum frequency, maximum frequency, and bandwidth) were inversely associated with forearm length. This may result from the fact that larger bats, possessing longer vocal tracts and larger larynges, tend to emit distress calls with lower spectral frequencies. This result is in line with previous research on birds and lizards, which documented a negative correlation between body size and frequency characteristics of distress calls [29,30,31]. Combined, morphology imposes a powerful pressure on the design of animal distress calls.

Owing to genetic inheritance of acoustic signals across generations [56], evolutionary conservatism in morphology or ecological niches which may determine vocal structures [19,55], animal vocalization characters usually contain reliable phylogenetic information. In herons, more similar vocal structure is exhibited between closely related species [64]. Similarly, a significant correlation between phylogenetic relatedness and acoustic similarity in the advertisement calls is found in a large number of anuran species [25]. Additionally, acoustic parameters of male vocalizations constitute plausible phylogenetic characters in 11 species of the Cervidae family [54]. In bats, phylogenetic relationships play a major role in shaping species-level divergence in duration and minimum frequency of aggressive calls [19]. Present results also indicated a robust relationship between phylogenetic components and distress calls structure in bats. More explicitly, phylogeny explained 58.18% interspecific variation in temporal parameters. Our findings therefore provide the first convincing evidence that distress calls in bats exhibit a strong phylogenetic signal.

Among the potential factors driving the interspecific divergence of vocalizations, the acoustic environment determining sound attenuation and degradation has received much attention [21,65]. Species occupying closed habitats should produce calls with lower frequencies to maximize signal broadcast and minimize atmospheric and vegetational absorption, scattering, and reverberation than open habitat species [65,66]. Similarly, low frequency sound tends to be favored in hot and humid conditions [21]. Additionally, previous studies documented a close link between social group size and vocal repertoire sizes and individual distinctiveness among different species, suggesting that sociality might act as an important driver of species-level acoustic divergence [28,67]. In this study, habitat features, climatic variables, and group size did not explain the interspecific variation in bat distress calls. Therefore, our results do not provide support for ecological selection and social pressure. As mentioned above, specific niche, flight and echolocation ability of bats insulate them from potential predators [51], and thus there is not strong pressure to promote the adaptive evolution in distress call structure to optimize signal propagation and vocal communication, and then individual survival. To confirm this, further work to test whether ecological and social factors play an important role in shaping distress vocalization in other taxa is required.

## 5. Conclusions

In summary, the evolutionary divergence of animal vocalization is a complex process shaped by various intrinsic and extrinsic factors. Distress calls, often used as indicators of emotional state and welfare in animals [68,69], also provide valuable insight into the evolutionary mechanisms underlying vocal behavior. However, less is known for the relative strength of different factors underlying cross-species acoustic divergence of distress calls, especially in mammals. Our results showed that bats divergence in distress vocalizations is not an adaptive evolution in response to ecological and social pressure, but constrained by phylogenetic relatedness and body size. This study expands our limited knowledge of the evolution of vocal behavior coping with distress in mammals.

## Figures and Tables

**Table 1:** Summary of strongly supported linear mixed models (ΔAICc ≤ 2) from model selection analysis.

Parameters	Models	Intercept	AICc	ΔAICc	*w*
Incidence of distress calls	PCO2	2.33	8.00	0.00	0.558
PC1 of acoustic variables	Forearm length + PCO1 + PCO2	2.81	78.70	0.00	0.218
	Forearm length + PCO1 + PCO2 + Colony size	1.66	79.20	0.53	0.167
	Forearm length + PCO1 + Colony size	1.53	79.60	0.940	0.136
	Forearm length + PCO1 + PCO2 + Annual Precipitation	3.45	80.60	1.86	0.086
PC2 of acoustic variables	PCO1 + PCO2 + Annual Precipitation	5.27	86.00	0.00	0.315
	PCO1 + PCO2	−4.22	86.70	0.69	0.223

Models are ranked according to their AICc values from the best to the worst. PCO1 and PCO2: the first and second phylogenetic component. AICc: Akaike information criteria corrected for sample size. ΔAICc: difference in AICc between each model and the best model. *w*: AICc weights.

**Table 2:** Factor loadings, eigenvalues, and percent of variance explained by principal component analysis performed on distress call parameters.

Measurement	PC1	PC2
Syllable rate (syllable/sec)	0.051	0.947
Syllable duration (ms)	−0.024	−0.944
Fundamental frequency (kHz)	0.912	0.08
Peak frequency (kHz)	0.925	−0.107
Minimum frequency (kHz)	0.89	−0.044
Maximum frequency (kHz)	0.925	0.102
Bandwidth (kHz)	0.777	0.144
Eigenvalue	3.962	1.818
% Variance explained	56.606	25.971

PC1 and PC2 represent the first and second principal component.

**Table 3:** Model-averaged parameter estimates from strongly supported linear models (ΔAICc ≤ 2) explaining interspecific variation in distress calls of bats, along with the independent effects of each predictor obtained from hierarchical partitioning analysis.

Predictor Variables	Parameters	PC1 of Acoustic Variables	PC2 of Acoustic Variables
Forearm length	RVI	**1.00**	−
	95% CI	**(−0.073, −0.037)**	−
	IE (%)	69.95 *	1.48
Colony size	RVI	**0.50**	−
	95% CI	**(0.051, 1.003)**	−
	IE (%)	11.54	3.72
Habitat type	RVI	−	−
	95% CI	−	−
	IE (%)	1.63	21.89
Annual mean temperature	RVI	−	−
	95% CI	−	−
	IE (%)	0.65	6.25
Annual precipitation	RVI	0.14	0.59
	95% CI	(−1.839, 1.414)	(−3.509, 0.125)
	IE (%)	0.65	2.53
Phylogenetic component 1	RVI	**1.00**	**1.00**
	95% CI	**(−5.423, −0.853)**	**(2.282, 7.612)**
	IE (%)	10.69	58.18 *
Phylogenetic component 2	RVI	0.78	**1.00**
	95% CI	(−7.057, 1.960)	**(2.392, 10.924)**
	IE (%)	4.90	5.95

Values in bold indicate predictor variables whose 95% confidence intervals did not overlap zero. RVI: relative variable importance. IE (%): independent effect (percentage of the explained variance) calculated using hierarchical partitioning analysis; asterisks denote statistical significance (* *p* < 0.05). A dash (−) indicates that the corresponding predictor variable was not retained in the strongly supported models explaining variation in acoustic parameters of distress calls.

## Data Availability

The data presented in this study are available from the corresponding author upon reasonable request.

## Supplementary Materials

The following supporting information can be downloaded at: https://www.mdpi.com/article/10.3390/ani15223268/s1, Figure S1: Representative spectrograms illustrating the acoustic parameters measured from bat distress calls; Figure S2: Principal component analysis (PCA) biplot of mean acoustic parameters across bat species; Table S1: Study sites and predictor variables; Table S2: Incidence of distress calls and acoustic parameters (Mean ± SD). References [19,40] are cited in the supplementary materials.
