# Supplementary material for: Phylogeny and Body Size Predict Distress Call Divergence in Bats: A Comparative Analysis
Source: Animals (Basel). 2025 Nov 12;15(22):3268. doi: 10.3390/ani15223268 (PMC12649752; doi:10.3390/ani15223268)
Supplement: Supplementary file 1 [file animals-15-03268-s001.zip › animals-3925461-supplementary.pdf]

Table S1. Study sites and predictor variables.

Species	Code	Location	Hab	Tem	N_{bats}	Pre	Size	PCO1	PCO2	PCO1'	PCO2'	FA	Accession N.
**Pteropodidae:**													
Cynopterus sphinx	*Cysp*	Xishuangbanna, Yunnan (BN)	N[40]	23.85	58	1559.19	400	-0.0324	-0.0857	-0.0389	0.0374	71.07±2.48	MG570060
Rousettus leschenaultii	*Role*	Dali, Yunnan (DL)	N[40]	23.82	9	1472.51	500	-0.0169	-0.1168	-	-	83.25±6.75	MG570061
**Hipposideridae:**													
Hipposideros cineraceus	*Hici*	Ningming, Guangxi (NM)	N[19]	23.29	19	1926.07	200	-0.0869	-0.0563	-0.0926	0.0223	35.31±0.66	KX467584
Hipposideros larvatus	*Hila*	Shaoguan, Guangdong (SG)	N[19]	24.47	15	2101.51	180	-0.0825	-0.0473	-0.0880	0.0284	59.36±1.76	MF630865
Hipposideros pratti	*Hipr*	Xixia, Henan (XX)	N[19]	16.71	8	1333.59	80	-0.0674	-0.0452	-0.0729	0.0223	87.33±3.22	MG570062
**Rhinolophidae:**													
Rhinolophus affinis	*Rhaf*	Lushi, Henan (LS)	N[19]	25.17	28	2526.91	300	-0.1309	0.0252	-0.1365	-0.0081	55.44±1.31	MG570063
Rhinolophus ferrumequinum	*Rhfe*	Linzhou, Hennan (LZ)	N[19]	13.42	32	653.61	120	-0.1252	0.0359	-0.1308	-0.0177	59.26±3.51	MG570064
Rhinolophus lepidus	*Rhle*	Jinning, Yunnan (JN)	N[19]	24.57	22	1377.73	500	-0.1289	0.0578	-0.1357	-0.0435	42.17±1.35	MF630875
Rhinolophus macrotis	*Rhmac*	Jinning, Yunnan (JN)	N[19]	18.63	27	1544.41	300	-0.1210	0.0450	-0.1278	-0.0281	43.51±1.00	KX467579
Rhinolophus marshalli	*Rhmar*	Hekou, Yunnan (HK)	N[19]	23.56	7	1557.50	70	-0.1305	0.0425	-0.1373	-0.0239	46.23±0.56	KX467580
Rhinolophus pearsonii	*Rhpe*	Anlong, Guizhou (AL)	N[19]	18.06	10	1433.65	110	-0.1174	0.0219	-0.1255	-0.0174	56.13±0.74	MG570065
Rhinolophus pusillus	*Rhpu*	Pingtan, Fujian (PT)	N[19]	20.71	22	1720.17	120	-0.1213	0.0148	-0.1279	0.0032	38.13±1.17	MG570066
Rhinolophus rex	*Rhre*	Jinning, Yunnan (JN)	N[40]	15.41	8	1177.69	50	-0.1281	0.0484	-0.1355	-0.0316	57.17±2.04	MG570067
Rhinolophus sinicus	*Rhsi*	Jinning, Yunnan (JN)	N[19]	16.49	32	1372.28	500	-0.1270	0.0078	-0.1338	0.0074	51.24±1.18	MF630879
**Vespertilionidae:**													
Eptesicus serotinus	*Epse*	Jinning, Yunnan (JN)	O[40]	7.59	3	490.39	20	-	-	0.1113	0.0495	52.35±0.91	MG570068
Pipistrellus alaschanicus	*Pial*	Jinan, Shandong (JIN)	E[19]	24.34	15	687.58	18	0.0816	-0.0849	0.0818	0.1269	33.88±1.56	KX467595
Ia io	*Iaio*	Xingyi, Guizhou (XY)	O[19]	17.72	18	1418.18	70	0.0724	0.0050	0.0727	-0.0053	74.77±1.18	MG570069
Murina leucogaster	*Mule*	Ji'an, Jilin (JA)	N[19]	15.11	23	1191.92	40	0.0888	-0.0142	0.0854	0.0210	42.87±0.91	KX467598
Myotis adversus	*Myad*	Liangqiu, Shandong (LQ)	E[19]	24.15	13	2492.46	1000	0.1199	0.0733	0.1145	-0.0855	41.07±1.44	KX467600
Myotis altarium	*Myal*	Zhashui, Shanxi (ZS)	N[19]	17.81	19	1408.51	400	0.1243	0.0144	0.1212	-0.0167	45.21±1.31	MF630871
Myotis badius	*Myba*	Jinning, Yunnan (JN)	**E**	146.40	24	1032.20	200	0.0370	0.0323	0.0317	-0.0335	36.09±1.34	MF630872
Myotis chinensis	*Mych*	Jiangyong, Hunan (JY)	N[19]	18.40	10	1450.71	500	0.1104	0.0210	0.1051	-0.0405	63.43±3.13	KX467607

Myotis fimbriatus	*Myfi*	Jinning, Yunnan (JN)	E[40]	14.54	14	1173.67	400	0.1050	0.0751	0.0992	-0.0726	42.62±0.63	MG570070
Myotis laniger	*Myla*	Jinning, Yunnan (JN)	E[19]	13.51	32	1171.60	500	0.0831	0.0520	0.0786	-0.0524	36.95±0.94	MF630874
Myotis macrodactylus	*Myma*	Ji’an, Jilin (JA)	E[19]	8.03	47	1298.70	500	0.1062	0.0780	0.1007	-0.0841	38.52±1.15	KX467603
Myotis pequinius	*Mype*	Jinan, Shandong (JIN)	N[19]	14.19	13	810.33	500	0.1160	0.0526	0.1088	-0.0781	50.47±0.98	KX467608
Myotis pilosus	*Mypi*	Liuan, Anhui (LA)	E[19]	17.45	15	1433.32	400	0.1117	0.0723	0.1062	-0.0828	56.12±1.70	MG570071
Pipistrellus abramus	*Piab*	Feixian, Shandong (FX)	E[19]	7.30	7	1122.52	20	0.0977	-0.0520	0.0942	0.0600	33.77±1.00	MG570072
Vespertilio sinensis	*Vesi*	Acheng, Heilongjiang (AC)	O[19]	3.91	27	754.85	500	0.0875	-0.0958	0.0878	0.1453	49.48±1.82	KX467594
**Miniopteridae:**													
Miniopterus fuliginosus	*Mifu*	Zhashui, Shanxi (ZS)	E[19]	13.80	23	638.62	1000	0.0038	-0.0939	-0.0004	0.0943	48.44±0.96	MG570073
**Molossidae:**													
Tadarida insignis	*Tain*	Jinning, Yunnan (JN)	**O**	14.80	3	1472.16	40	-	-	0.0163	0.0411	62.13±0.68	MG570074
**Emballonuridae:**													
Taphozous melanopogon	*Tame*	Puer, Yunnan (PE)	O[19]	24.35	20	1864.50	120	0.0710	-0.0834	0.0679	0.0625	64.42±2.46	MG570075

Hab: N narrow habitat, E edge habitat, O open habitat. N_{bats}: the number of bats caught. Tem: annual mean temperature. Pre: annual precipitation. Size: estimated colony size. PCO1 and PCO2: the first 2 eigenvectors of the genetic distances omitting *E. serotinus* and *T. insignis* extracted via principal coordinate function (PCoA). PCO1’ and PCO2’: the first 2 eigenvectors of the genetic distances omitting *R. leschenaultii* extracted via principal coordinate function (PCoA). Accession N.: GenBank accession number. FA: Forearm length (mm). The values are shown with mean ± SD for FA. Regarding foraging habitat, data in bold come from field survey and others are sourced from the following reference.

[19] Luo, B.; Huang, X.; Li, Y.; Lu, G.; Zhao, J.; Zhang, K.; Zhao, H.; Liu, Y.; Feng, J. Social call divergence in bats: a comparative analysis. *Behav. Ecol.* **2017**. 28:533-540.

[40] IUCN, The IUCN red list of threatened species. Version **2023-2**. Available at: http://www.iucnredlist.org. (Accessed on 13 May 2024).

Table S2. Incidence of distress calls and acoustic parameters (Mean ± SD).

Species	N_{bats}	N_{caller}	% caller	$N_{analysis}$	Rate	Duration	F_{fun}	F_{peak}	F_{min}	F_{max}	Band
Cysp	58	37	63.79	15	3.25 ± 0.88	253.42 ± 58.79	7.29 ± 0.99	7.29 ± 0.99	4.60 ± 0.28	26.00 ± 4.43	21.34 ± 4.36
Role	9	0	0.00	0	-	-	-	-	-	-	-
Hici	19	15	78.95	13	10.64 ± 1.30	76.71 ± 11.81	51.19 ± 3.71	51.54 ± 3.83	31.39 ± 8.05	91.52 ± 11.26	60.09 ± 12.34
Hila	15	13	86.67	13	10.25 ± 5.26	58.31 ± 17.49	13.27 ± 2.57	13.52 ± 2.55	6.47 ± 0.53	70.11 ± 12.64	63.58 ± 12.35
Hipr	8	6	75.00	6	7.76 ± 2.99	85.16 ± 19.94	10.69 ± 2.76	10.98 ± 3.03	5.88 ± 1.59	53.13 ± 12.01	47.42 ± 11.26
Rhaf	28	28	100.00	14	4.96 ± 0.79	157.08 ± 29.09	28.41 ± 3.06	34.71 ± 5.60	18.80 ± 3.03	86.26 ± 4.61	67.41 ± 5.22
Rhfe	32	32	100.00	21	5.03 ± 0.94	155.47 ± 33.39	26.22 ± 1.76	37.05 ± 8.99	20.66 ± 2.02	79.07 ± 9.98	58.37 ± 10.47
Rhle	22	22	100.00	15	5.53 ± 0.88	147.74 ± 31.25	25.58 ± 2.12	43.63 ± 6.94	17.37 ± 1.53	96.79 ± 8.43	79.38 ± 8.16
Rhmac	27	27	100.00	17	6.63 ± 1.25	123.56 ± 40.27	17.56 ± 1.39	47.43 ± 6.26	13.58 ± 1.03	70.13 ± 3.06	56.49 ± 3.53
Rhmar	7	7	100.00	7	5.90 ± 0.41	128.27 ± 8.91	13.99 ± 1.16	25.82 ± 5.26	8.35 ± 1.21	54.36 ± 2.41	45.95 ± 2.88
Rhpe	10	9	90.00	9	6.09 ± 0.49	113.69 ± 19.38	11.48 ± 1.52	23.08 ± 8.58	7.66 ± 1.13	68.28 ± 4.48	60.56 ± 3.94
Rhpu	22	22	100.00	19	6.46 ± 1.24	131.44 ± 41.51	27.65 ± 4.15	34.09 ± 5.13	13.69 ± 2.98	104.79 ± 13.60	91.05 ± 13.61
Rhre	8	8	100.00	8	5.32 ± 0.47	122.49 ± 24.42	16.18 ± 1.84	22.78 ± 1.47	7.21 ± 0.56	35.66 ± 2.02	28.4 ± 2.24
Rhsi	32	32	100.00	19	5.31 ± 0.69	155.58 ± 22.77	29.40 ± 1.68	38.87 ± 6.38	20.47 ± 2.17	81.51 ± 5.23	61.00 ± 6.5
Epse	3	2	66.67	2	14.33 ± 3.98	34.55 ± 2.33	22.17 ± 4.82	22.40 ± 4.48	12.38 ± 2.65	60.55 ± 8.07	48.12 ± 5.45
Pial	15	12	80.00	11	5.79 ± 1.68	101 ± 32.15	19.08 ± 2.26	20.4 ± 2.56	10.23 ± 1.15	67.97 ± 7.13	57.69 ± 7.49
Iaio	18	18	100.00	18	6.76 ± 0.90	69.16 ± 11.84	10.71 ± 1.87	10.91 ± 1.80	7.28 ± 1.05	35.38 ± 6.21	28.05 ± 5.83
Mule	23	16	69.57	9	10.85 ± 2.82	65.79 ± 22.97	27.5 ± 4.79	29.37 ± 7.12	13.94 ± 4.04	74.79 ± 17.11	60.81 ± 14.19
Myad	13	11	84.62	7	8.78 ± 1.73	72.54 ± 14.88	26.99 ± 3.70	27.40± 3.79	11.52 ± 3.96	85.00 ± 15.41	73.42 ± 12.99
Myal	19	19	100.00	15	6.81 ± 1.10	91.72 ± 19.74	24.69 ± 2.50	39.32 ± 4.19	9.95 ± 0.85	71.08 ± 4.01	61.07 ± 4.11
Myba	24	23	95.83	17	6.97 ± 1.41	95.31 ± 19.75	42.55 ± 5.03	42.76 ± 4.71	22.47 ± 5.53	74.74 ± 8.68	52.22 ± 10.90
Mych	10	8	80.00	8	11.29 ± 3.41	50.82 ± 36.7	9.10 ± 1.33	11.09 ± 1.89	5.66 ± 0.29	59.01 ± 8.32	53.29 ± 8.33
Myfi	14	14	100.00	11	7.54 ± 1.39	79.87 ± 19.09	30.47 ± 4.74	32.39 ± 5.96	14.81 ± 3.61	78.88 ± 2.79	64.03 ± 4.51
Myla	32	32	100.00	12	6.83 ± 1.34	102.21 ± 24.05	37.87 ± 2.13	38.85 ± 2.92	23.75 ± 3.43	76.61 ± 5.18	52.81 ± 6.17
Myma	47	32	68.09	12	6.85 ± 2.67	99.34 ± 45.60	29.70 ± 3.12	31.31 ± 3.92	17.81 ± 2.82	71.19 ± 3.97	53.33 ± 4.52
Mype	13	11	84.62	11	4.31 ± 1.16	158.31 ± 44.53	17.2 ± 1.70	17.72 ± 1.52	8.8 ± 1.19	51.12 ± 8.79	42.26 ± 9.09

Mypi	15	14	93.33	11	8.13 ± 1.64	77.89 ± 28.47	21.03 ± 3.71	23.45 ± 3.79	8.34 ± 1.25	72.32 ± 3.84	63.94 ± 3.56
Piab	7	5	71.43	4	4.38 ± 0.73	163.15 ± 43.82	22.46 ± 1.12	22.95 ± 1.31	12.08 ± 2.24	64.89 ± 7.41	52.76 ± 7.82
Vesi	27	27	100.00	17	9.12 ± 4.43	88.23 ± 37.69	11.23 ± 1.18	11.34 ± 1.25	7.95 ± 0.58	47.72 ± 6.39	39.71 ± 6.73
Mifu	23	22	95.65	17	10.00 ± 1.23	55.29 ± 10.53	36.01 ± 2.66	37.25 ± 3.51	17.77 ± 3.71	86.85 ± 5.38	69.04 ± 7.30
Tain	3	3	100.00	3	7.43 ± 0.55	47.01 ± 3.86	8.02 ± 0.52	9.36 ± 1.00	6.47 ± 0.31	25.45 ± 4.74	18.90 ± 4.48
Tame	20	9	45.00	7	8.28 ± 11.77	152.56 ± 63.95	11.30 ± 1.31	14.16 ± 5.07	5.90 ± 0.49	52.51 ± 6.96	46.55 ± 7.04

N_{bats}: the number of bats caught. N_{caller}: the number of bats producing distress calls while being handled. $N_{analysis}$: the number of bats used for acoustic analysis. Rate: syllable rate (syllable/sec). Duration: syllable duration (ms). F_{fun}: fundamental frequency (kHz). F_{peak}: peak frequency (kHz). F_{min}: minimum frequency (kHz). F_{max}: maximum frequency (kHz). Band: bandwidth (kHz). For full name of the species, please see Table S1.

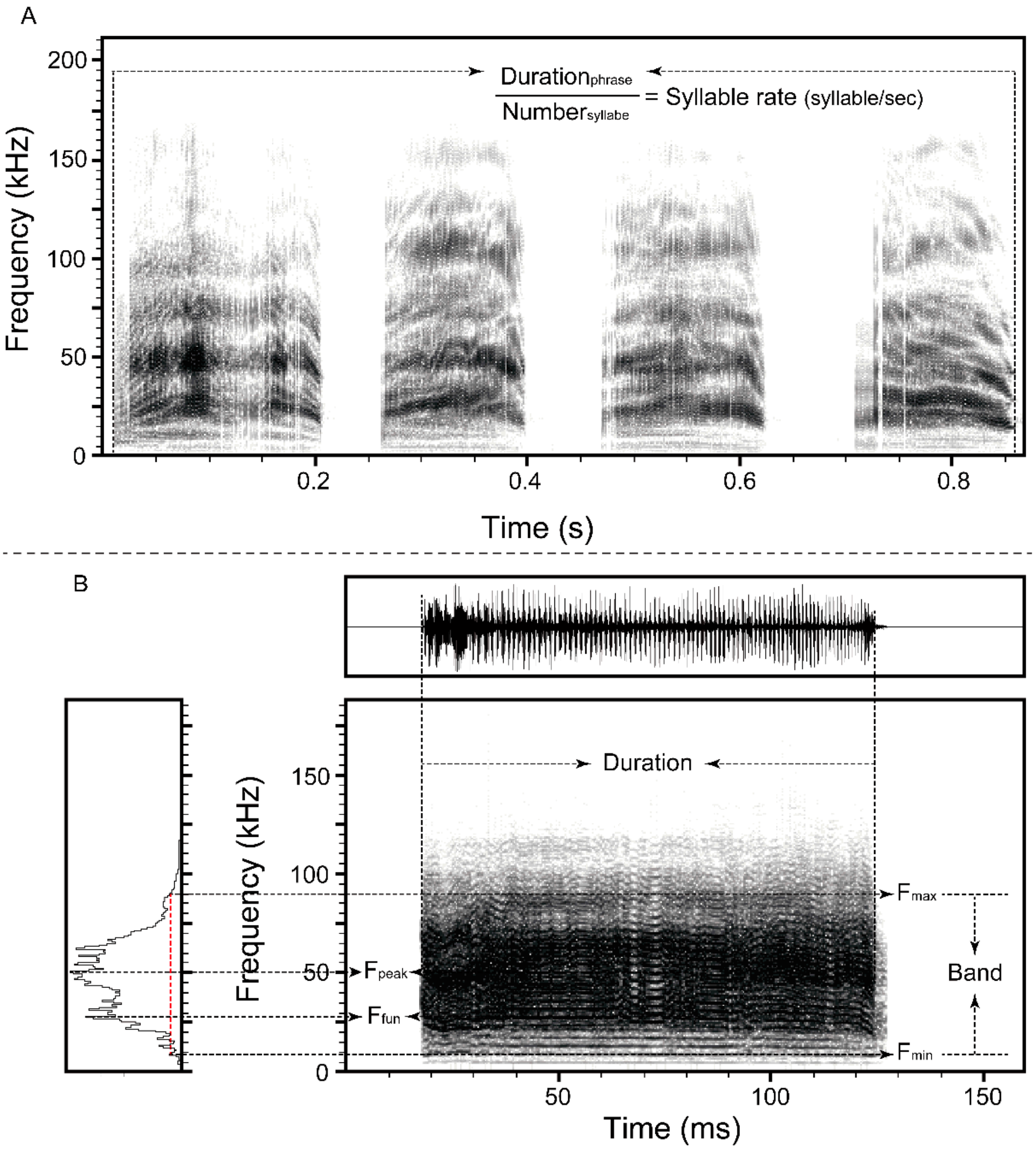

Figure S1. Representative spectrograms illustrating the acoustic parameters measured from bat distress calls. (A) Measurement of syllable rate (syllable/sec). (B) Measurement of syllable duration (Duration, ms), fundamental frequency (F_{fun}, kHz), peak frequency (F_{peak}, kHz), minimum frequency (F_{min}, kHz), maximum frequency (F_{max}, kHz), and bandwidth (Band, kHz). $Duration_{phrase}$: phrase duration; $Number_{syllable}$: number of syllables within a phrase.

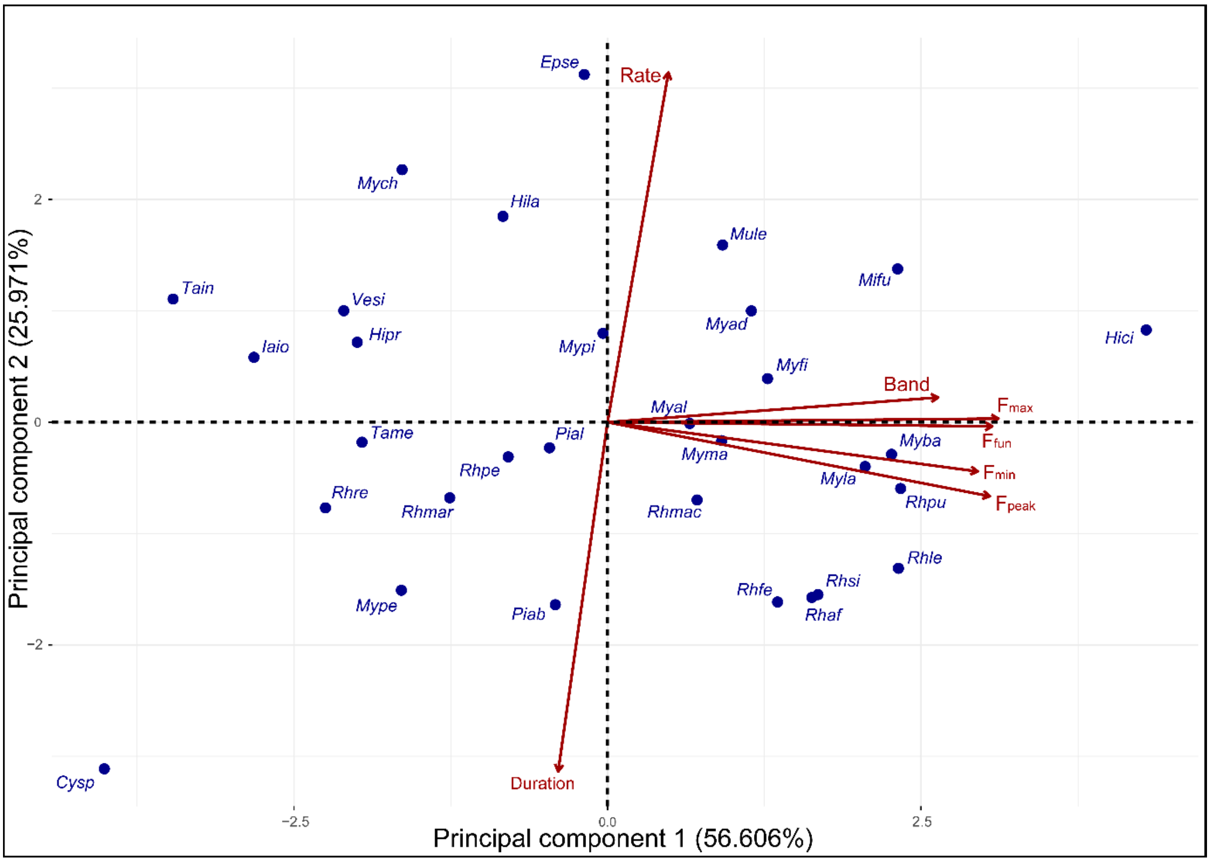

Figure S2. Principal component analysis (PCA) biplot of mean acoustic parameters across bat species. This plot shows the first two principal components (PC1 and PC2) derived from the PCA of mean acoustic parameters across bat species. Blue points represent different bat species, and red arrows indicate the direction and magnitude of the contributing acoustic parameters. The percentage values on the axes (PC1: 56.606%, PC2: 25.971%) represent the variance explained by each component. Full names of all abbreviations are provided in Tables S1 and S2.
